# The diagnostic threshold of Cornell assessment of pediatric delirium in detection of postoperative delirium in pediatric surgical patients

**DOI:** 10.1186/s12887-021-02538-x

**Published:** 2021-02-17

**Authors:** Hong Hong, Chao Guo, Zhi-Hua Liu, Bo-Jie Wang, Shu-Zhe Zhou, Dong-Liang Mu, Dong-Xin Wang

**Affiliations:** 1 Department of Anesthesiology and Critical Care Medicine, Peking University First Hospital, No. 8, Xi-Shi-Ku Street, Xi Cheng District, 100034 Beijing, China; 2 Department of Psychiatry, Peking University Sixth Hospital (Institute of Mental Health), No. 51, Hua-Yuan Road, Hai Dian District, 100191 Beijing, China

**Keywords:** Cornell assessment of pediatric delirium, Chinese version, Pediatric delirium, Surgery, Threshold

## Abstract

**Background:**

Cornell assessment of pediatric delirium (CAPD) showed advantage in diagnosis of pediatric delirium in Chinese critically ill patients. But its performance in surgical patients is still unclear. The present study was designed to validate the diagnostic performance of CAPD in surgical pediatric patients.

**Methods:**

This is a prospective validation study. Pediatric patients who underwent selective surgery and general anesthesia were enrolled. Primary outcome was the incidence of delirium within postoperative three days. CAPD Chinese version was used to evaluate if the patient had delirium one time per day. At the meantime, a psychiatrist employed Diagnostic and Statistical Manual of Mental Disorders fifth edition to diagnose delirium, which was the “gold standard”, and the result was considered as reference standard. Sensitivity, specificity and area under receiver operating characteristic (ROC) curve were calculated to investigate the performance of CAPD.

**Results:**

A total of 170 patients were enrolled. Median age was 4 years old. As diagnosed by psychiatrist, 23 (13.5 %) patients experienced at least one episode of delirium during the follow-up period. When diagnostic threshold was set at 9, CAPD showed the optimal sensitivity (87.0 %, 95 %CI 65.3 %-96.6 %) and specificity (98.0 %, 95 %CI 93.7 %-99.5 %) in comparison with other diagnostic thresholds. ROC analysis showed that CAPD was a good delirium assessment instrument with area under curve of 0.911 (95 % CI 0.812 to 1.000, *P* < 0.001). Agreement between CAPD and reference standard was 0.849 (Kappa coefficient, *P* < 0.001).

**Conclusions:**

This study found that Cornell assessment of pediatric delirium could be used as an effective instrument in diagnosis of delirium in pediatric surgical patients.

**Trial registration:**

www.chictr.org.cn Identifier: ChiCTR-DDD-17,012,231, August 3, 2017.

## Background

Pediatric delirium is an acute brain dysfunction which is characterized by acute change or fluctuation of mental status, inattention, altered level of consciousness and neurobehavioral dysfunction [1]. The prevalence of pediatric delirium varies from 12 to 28 % in critically ill patients [1, 2]. In surgical patients, its incidence reaches as high as 66 % [3, 4]. Delirium is associated with increased medical cost and poor clinical outcome such as increased mortality [5, 6].

Key barrier in pediatric delirium is the difficulty of diagnosis, because immature development of the child limits the accuracy and feasibility of neuropsychological assessment [7]. To facilitate the diagnosis, several brief instruments had been developed including pediatric confusion assessment method for intensive care unit (pCAM-ICU), pediatric anesthesia emergence delirium (PAED), and Cornell assessment of pediatric delirium (CAPD) [1–3, 8]. CAPD has two advantages in comparison with other instruments. First, it can be used in children of all ages. pCAM-ICU is applicable to the evaluation of children aged 5 years or above while PAED is applicable to patients aged from 19 months to 6 years [1, 3]. Second, CAPD is the most promising and available tool to detect hypoactive delirium [2, 8]. The accuracy and efficacy of CAPD Chinese version have been validated in pediatric patients in intensive care unit (ICU) [9]. When cut-off point was set at 10, CAPD presented excellent performance (sensitivity 96.7 % and specificity 93.1 %) and high inter-rater agreement (Kappa = 0.835) [9].

Several factors might affect the performance of neuropsychological instrument. First, the diagnostic characteristic of the instrument might show discrepancy in different populations (i.e., surgical patients versus critically ill patients) [10]. Second, sampling bias and limited sample size in each study might affect the accuracy and efficacy estimate of instrument. Thus, it’s necessary to further investigate the diagnostic performance of CAPD in different groups. For example, risk factors of delirium in surgical and ICU patients are different [11]. Pain is one of the main causes of postoperative delirium, whereas mechanical ventilation mainly contributes to delirium in ICU [11, 12].

This study was designed to investigate the diagnostic threshold of Cornell assessment of pediatric delirium in detection of delirium in surgical patients.

## Methods

 This prospective validation study was approved by Peking University First Hospital institution review board (No. 2017 − 1344) and registered at Chinese Clinical Trial Registry on August 3, 2017. Written inform was obtained from patients’ parents or their legal surrogates. The study was conducted in a tertiary teaching hospital from November 2017 to December 2018.

### Participants

Pediatric patients who underwent selective surgery were screened [8]. Eligible patients were enrolled if they received general anesthesia and expected postoperative in-hospital stay was more than 48 hours. Patients were excluded if they met one of the following criteria: (1) unable to complete delirium assessment, i.e., severe cognitive dysfunction, coma and deep sedation; (2) severe visual or hearing impairment which impeded delirium assessment.

### Delirium assessment

Primary outcome was the incidence of delirium within postoperative 3 days. Patient was visited at 16:00–18:00 every day. Delirium assessment was completed by an anesthesiologist (ZH-L) and a psychiatrist (SZ-Z) within 30 minutes interval. During the study period, they were blinded to the assessment result of each other.

### Training of CAPD application

CAPD had been translated into Chinese version and validated in critically ill patients by Dr. He and colleagues [9]. We employed the Chinese version to diagnose delirium in this study. Before the beginning of the study, all researchers received a training session including four parts: (1) introduction of study protocol; (2) lecture on the clinical symptoms, signs and diagnosis of pediatric delirium by psychiatry expert; (3) introduction of CAPD and its application; and (4) simulation training courses on patient-actors until the diagnosis of delirium reached 100 % agreement between researchers.

### Gold standard

Psychiatrist (SZ-Z) employed Diagnostic and Statistical Manual of Mental Disorders fifth edition (DSM-5) to evaluate if children suffered delirium [13]. This was considered as gold standard to calculate the diagnostic performance of CAPD.

### Anesthesia and surgery

All patients received surgery under general anesthesia. Propofol and remifentanil (with or without sufentanil) were used for anesthesia induction and maintenance. Nitrous dioxide and sevoflurane could be used as supplementation to general anesthesia in necessary. Muscle relaxant was maintained by intermittent injection of cisatraurium. Routine intraoperative monitoring included electrocardiogram, non-invasive blood pressure, pulse oxygen saturation and end tidal carbon dioxide. Anesthetic depth was adjusted to maintain Bispectral Index between 40 and 60. After surgery, patients were transferred to post-anesthesia care unit (PACU) for recovery and discharged when Alderet score reached 9 or above [14].

### Statistical analysis

### Sample size

The incidence of delirium in surgical pediatric patients varied from 18 %, 25 % and 66 % in different types of surgery [4, 15, 16]. We assumed the average incidence of postoperative delirium was 20 %. Based on our previous study [17], the width of confidence interval was set at 0.1. If the expected sensitivity and specificity were set at 0.9 respectively, significance level at 0.05, according to the equation below[18], we needed 163 patients. Considering a 5 % of follow-up loss, 170 patients were enrolled.

Sample size (n) based on sensitivity:

$$ \frac{{Z^2}_{1-a/2}\times {S}_N\times \left(1-{S}_N\right)}{L^2\times Prevalence} $$

sample size (n) based on specificity:

$$ \frac{{Z^2}_{1-a/2}\times {S}_P\times \left(1-{S}_P\right)}{L^2\times \left(1- Prevalence\right)} $$

*S*_*N*_ = anticipated sensitivity, *S*_*P*_ = anticipated specificity, α = size of the critical region (1−α is the confidence level), Z_1-α/2_= standard normal deviate corresponding to the specified size of the critical region (α), and L = absolute precision desired on either side (half-width of the confidence interval) of sensitivity or specificity.

### Outcome analysis

In general principle, continuous data with non-normal distribution was presented as median (interquartile), and categorical variables were presented as number of patients (percentage). Sensitivity and specificity were analyzed to test the diagnostic performance of CAPD [19]. The receiver operating characteristic (ROC) curve, along with the area under the curve and 95 % confidence interval (CI), was utilized to assess the ability of different CAPD thresholds to diagnose delirium.

P value less than 0.05 was considered as statistical significance. All statistical analyses were performed with the SPSS statistical package version 14.0 (SPSS Inc, Chicago, Ill) and R (version 3.6.3; R Development Core Team, Vienna, Austria).

## Results

### Patients

**Fig. 1:**
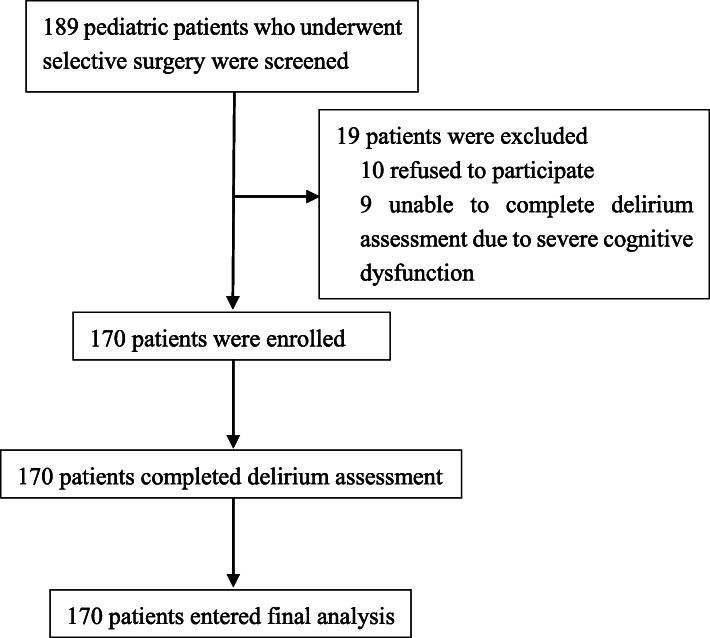
Flowchart

During the study period, 189 patients were screened and 170 eligible patients were enrolled, Fig. 1. Patient’s age ranged from 1 month to 18 years old and median age was 4 years old, Table 1.

**Table 1:** Baseline characteristic of patients

Variables	All patients (*N* = 170)	Delirious patients (*N* = 23)	Non-delirious patients (*N* = 147)	P
Age, median (IQR), months	48 (25.5, 84.0)	36 (24.0, 72.0)	48 (36.0, 90.0)	0.071
Age group, n (%)				0.594
0–6 months, n (%)	7 (4.1)	1 (4.3)	6 (4.1)	
6 months-2 years, n (%)	35 (20.6)	7 (30.4)	28 (19.0)	
2–5 years, n (%)	61 (35.9)	6 (26.1)	55 (37.4)	
≥5 years, n (%)	67 (39.4)	9 (39.1)	58 (39.5)	
Female, n (%)	59 (34.7)	4 (17.4)	55 (37.4)	0.061
Height, median (IQR), cm	110 (95.0, 130.0)	105 (87.0, 116.0)	110 (96.0, 130.0)	0.049
Body weight, median (IQR), kg	19.0 (14.9, 28.3)	16.0 (12.0, 21.0)	19.3 (15.0, 29.0)	0.033
Previous medical history				
Pre-term, n (%)	15 (8.8)	2 (8.7)	13 (8.8)	>0.999
Epilepsy, n (%)	2 (1.1)	0	2 (1.4)	>0.999
Congenital heart disease, n (%)	2 (1.1)	0	2 (1.4)	>0.999
Surgery type, n (%)				0.001
Ear-nose-throat surgery	77 (45.3)	6 (26.1)	71 (48.3)	
General surgery	41 (24.1)	3 (13.0)	38 (25.9)	
Urological surgery	29 (17.1)	4 (17.4)	25 (17.0)	
Neurosurgery	23 (13.5)	10 (43.5)	13 (8.8)	
Surgery time, median (IQR), min	47.0 (29.5, 72.0)	57.0 (27.0, 97.0)	47.0 (29.5, 71.0)	0.279
Anesthesia time, median (IQR), min	100.0 (83.0, 130.5)	110.0 (79.0, 162.0)	100.0 (83.0, 129.5)	0.347
Postoperative LOS, median (IQR), day	3 (1, 5)	4 (1, 10)	3 (1, 5)	0.114

*IQR *interquartile range, *LOS *length of in-hospital stay

### Incidence of delirium

**Fig. 2:**
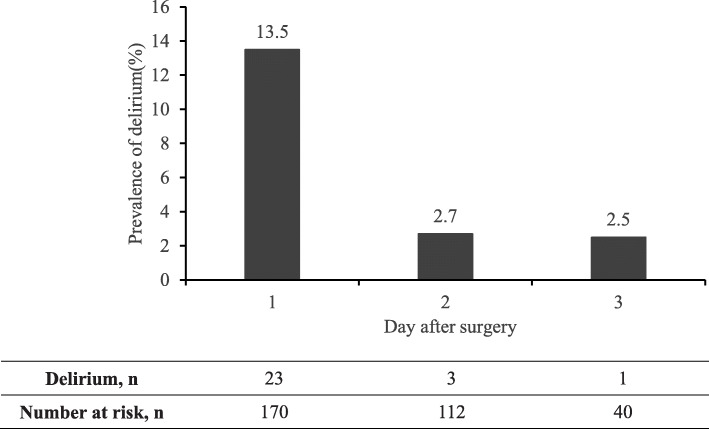
Daily prevalence of delirium

**Fig. 3:**
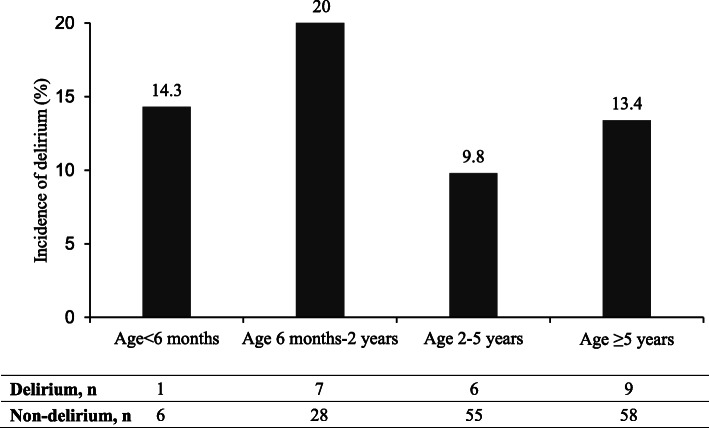
Incidence of delirium at different age groups

Delirium assessments were conducted after surgery until the 3rd postoperative day or discharge. During the study period, a total of 322 paired assessments (anesthesiologist-psychiatrist) was completed, including 170 on the first postoperative day, 112 and 40 on the second and third day respectively. As diagnosed by psychiatrist, 23 (13.5 %) patients experienced 27 episodes of delirious events in total, and 85.2 % (23/27) was on the first day after surgery. Daily prevalence of delirium was 13.5 % (23/170), 2.7 % (3/112), 2.5 % (1/40) from first to third postoperative day respectively, Fig. 2. Distribution of delirium at different age groups was depicted in Fig. 3, i.e., 20.0 % (7/35) in patient with age between 6 months and 2 years. The incidence of delirium was about 7.8 % (6/77) after ear-nose-throat surgery, 7.3 % (3/41) after general surgery, 13.8 % (4/29) after urological surgery and 43.5 % (10/23) after neurosurgery respectively, Table 1.

### Diagnostic character of CAPD

Calculation of diagnostic performance of CAPD was based on 170 paired assessments on the postoperative first day. When diagnostic score of CAPD was set at 9, 10, and 11, the incidence of delirium was 13.5 % (23/170), 10.6 % (18/170) and 9.4 % (16/170) respectively.

**Fig. 4:**
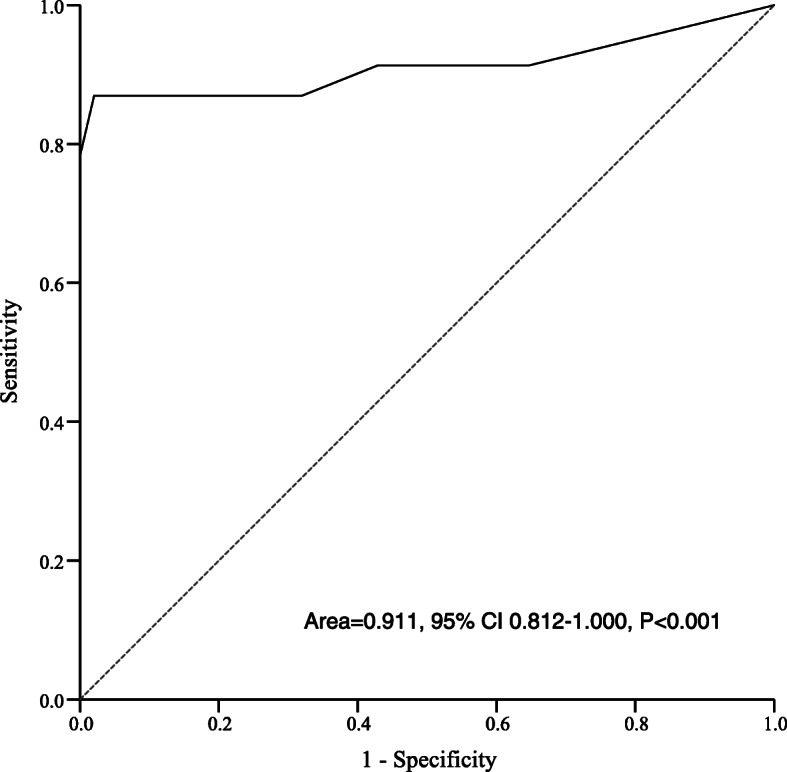
Receiver operating characteristic curve

When diagnostic threshold was set at 9, CAPD showed the optimal sensitivity (87.0 %, 95 %CI 65.3 %-96.6 %) and specificity (98.0 %, 95 %CI 93.7 %-99.5 %) in comparison with diagnostic threshold at 10 or 11, Table 2. The agreement between CAPD (threshold at 9) and reference standard was 0.849 (Kappa coefficient, *P* < 0.001). ROC analysis showed that CAPD could be used as a good instrument for postoperative delirium assessment with area under curve of 0.911 (95 % CI 0.812-1.000, *P* < 0.001), Fig. 4.

**Table 2:** Diagnostic performance of Cornell assessment of pediatric delirium

	CAPD score ≥ 9		CAPD score ≥ 10		CAPD score ≥ 11	
**Reference standard**	Delirium (n)	Non-delirium (n)	Delirium (n)	Non-delirium (n)	Delirium (n)	Non-delirium (n)
**Delirium** (n)	20	3	18	5	16	7
**Non-delirium** (n)	3	144	0	147	0	147
**Sensitivity (95 % CI)**	87.0 % (65.3 %-96.6 %)		78.3 % (55.8 %-91.7 %)		69.6 % (47.0 %-85.9 %)	
**Specificity (95 % CI)**	98.0 % (93.7 %-99.5 %)		100 %		100 %	
**Kappa coefficient**	0.849		0.862		0.798	
**P**	< 0.001		< 0.001		< 0.001	

## Discussion

This study demonstrated that Cornell assessment of pediatric delirium could be used as an effective instrument in diagnosis of delirium in pediatric surgical patients.

Pediatric delirium has arisen intensive concern in recent years because of its high prevalence and significant adverse effect on patients’ clinical outcome [4, 11]. Therapeutic methods have been investigated to reduce delirium and its adverse effect [20]. However, these medical care or interventions depend on the correct diagnosis of delirium [20].

Delirium mainly manifested as neuropsychological (i.e., cognitive impairment and altered consciousness) dysfunction and behavioral disorder. In adult patients, it’s relatively easy to evaluate cognitive function and consciousness level by neuropsychological scales [21]. Children are at underdevelopment of nervous system, so the diagnosis of delirium in these patients focuses dominantly on behavioral changes rather than cognitive impairment in adult [21]. Taking cognition as an example, it can be tested by Mini-cog or Mini-Mental State Examination in adult [21]. However, for infants or young children, they cannot report or illustrate their discomfort or disability in accurate expressions [22]. In CAPD, behaviors including eye contact with caregivers and purposeful action were used to detect inattention and cognitive dysfunction [3].

The major finding of this study showed that the optimal threshold for CAPD to diagnose delirium in surgical pediatric patients was 9, which was different from that in critically ill patients in previous study [9]. It’s not surprising to find that neuropsychological instruments have different threshold in diverse groups [10]. First, the composition of patients was different. Patients aged < 2 years were reported at increased risk of delirium [11, 20]. The percentage of young children (age < 2 years) was about 78.3 % [9] in Dr. He and colleague’s study but 24.7 % of enrolled patients in this study. Second, the severity of illness differs. Up to 83.6 % of critically ill patients received mechanical ventilation during the stay of ICU [9]. However, there was no ICU admission after surgery in this study.

We also noticed that the diagnostic performance of CAPD in Chinese patients was lower than that in the English-speaker patients [8, 9]. This phenomenon is reasonable as cross-culture translation of neuropsychological instrument which may decrease its sensitivity and specificity [10]. For example, one item of CAPD is “Is the child inconsolable?” which needs the involvement of parents to alleviate children’s anxiety. In China, parents are not allowed to accompany their children in the PACU or ICU in most medical centers. Thus, the incidence of “inconsolable” may be increased and leading to false “positive diagnosis”.

Several brief instruments had been developed for assessment of pediatric delirium. PAED was widely used in perioperative settings to access agitation and delirium and it’s also the basis of CAPD [3]. PAED focuses on patients with hyperactive symptoms which may underestimate the incidence of hypoactive delirium [2, 3, 23]. Both p-CAM-ICU and preschool-CAM-ICU were derived from confusion assessment method for ICU [1, 24]. In critically ill children of 5 years old or older, it’s reported that p-CAM-ICU showed better test validity in comparison with PAED [25]. But there is lack of sufficient data to compare the performance of CAPD with other instruments.

The present study had two limitations. First, this is a single center cohort study and the result should be validated in multicenter with larger sample size. Second, the CAPD should be adapted according to Chinese culture to improve its diagnostic performance.

## Conclusions

The main finding of the present study was that Cornell assessment of pediatric delirium could be used as an effective instrument in diagnosis of delirium in pediatric surgical patients. When cutoff point was set at 9, the instrument showed the best diagnostic performance. This result provides strong evidence to facilitate delirium assessment in surgical pediatric patients. As a single center cohort study, our result needs to be further verified by multicenter trials.

## Data Availability

The datasets used and/or analyzed during the current study are available from the corresponding author on reasonable request.

## Abbreviations

CAPD: Cornell assessment of pediatric delirium
CI: Confidence interval
ICU: :Intensive care unit
pCAM-ICU: Pediatric confusion assessment method for intensive care unit
PAED: Pediatric anesthesia emergence delirium
DSM-5: Diagnostic and Statistical Manual of Mental Disorders fifth edition
PACU: Post-anesthesia care unit
ROC: Receiver operating characteristic
